# Identification and Validation of an m7G-Related lncRNAs Signature for Prognostic Prediction and Immune Function Analysis in Endometrial Cancer

**DOI:** 10.3390/genes13081301

**Published:** 2022-07-22

**Authors:** Jiani Sun, Li Li, Hong Chen, Lei Gan, Xiaoqing Guo, Jing Sun

**Affiliations:** Department of Gynecology, Shanghai First Maternity and Infant Hospital, School of Medicine, Tongji University, Shanghai 200092, China; sunjiani5947@163.com (J.S.); 1931199@tongji.edu.cn (L.L.); chenhong377@163.com (H.C.); gan_leilei@126.com (L.G.); xiaoqing_guo@tongji.edu.cn (X.G.)

**Keywords:** N7-methylguanosine, long noncoding RNAs, endometrial carcinoma, prognostic model, immune function

## Abstract

Background: N7-methylguanosine is a novel kind of internal modification that is widespread in human mRNA. The relationship between m7G-related lncRNAs (MRL) and endometrial cancer remains unknown. The aim of our study is to explore a predictive prognosis MRL signature in endometrial cancer and identify the underlying biological mechanism. Methods: We obtained RNA-seq profiles, clinical data, and information on somatic mutations from the TCGA database and obtained m7G-related genes from a previous study. MRLs were identified through a co-expression network. The prognostic model was constructed based on 10 m7G-related lncRNAs. Differentially expressed genes between low- and high-risk groups were identified for further analysis, consisting of functional enrichment analysis, immune function analysis, somatic mutation analysis, and potential drugs exploration. Results: We constructed a 10-MRLs signature. According to the risk score, the signature was classified into high- and low-risk groups. The signature had a reliable capacity for predicting the prognosis of endometrial cancer patients. The findings about differentially expressed genes were also of great significance for therapeutic treatments for endometrial cancer and gave novel insights into exploring the underlying molecular mechanism. Conclusion: The prognostic model based on 10 MRLs is a reliable and promising approach for predicting clinical outcomes and suggesting therapeutic methods for endometrial cancer patients.

## 1. Introduction

Uterine corpus endometrial carcinoma (UCEC) is the most common cancer of the female reproductive system worldwide, ranking second among female genital cancers, with a mortality rate of 2.7 per 100,000 in China [1,2]. Depending on etiology and clinical variables, UCEC is generally classified as type I endometrioid EC or type II non endometrioid EC [3]. Most endometrial cancers (72%) are detected in the early stage (stage I/II), whereas 20% have regional metastasis (stage III), and 8% have distant metastasis (stage IV) [4]. Although most endometrial cancers are diagnosed at early and treatable stages, late diagnosis of endometrial cancers at advanced stages remains challenging to treat. At present, UCEC are treated primarily by hysterectomy in the early stage, and with surgery and other adjuvant therapies in the advanced stage [5]. However, advanced UCEC, which is characterized by highly aggressive and easily metastatic clinical behavior, shows poor outcomes. Although it is highly curable by surgery when diagnosed at an early stage and grade, conventional surgery brings devastating consequences for fertility to these patients [6]. Therefore, it is of great significance for the improvement of early diagnosis and treatment of UCEC to understand the molecular mechanisms and explore new potential biomarkers, as well as promising therapeutic targets.

Long noncoding RNAs (lncRNAs) are regulatory RNA transcripts longer than 200 nucleotides without coding capacity [7]. Long noncoding RNAs have emerged as potent regulators of gene expression at different levels, including chromatin remodeling, transcriptional and post-transcriptional control, and protein metabolism [8]. A large number of studies have indicated that lncRNAs are highly associated with the progression of a wide variety of diseases through their crosstalk with other macromolecules, including DNA, RNA, and protein [9,10]. The accumulated evidence has demonstrated that lncRNAs are frequently dysregulated in cancers, and are involved in the progression and metastasis of multiple malignancies. It has been reported that lncRNAs play a pivotal role in the development and progression of cancer, and might function as cancer biomarkers and novel therapeutic targets.

Recent discoveries have highlighted the regulatory roles of RNA modification in the control of gene expression regulation and the course of cancer. To date, over 160 RNA modifications have been identified, including N7-methylguanine (m7G), N6-methyladenosine (m6A), and 5-methylcytosine (m5C) [11]. It is known that m7G is one of the most abundant modifications present in tRNA, rRNA, and mRNA 5′cap, and plays an essential role in regulating multiple aspects of RNA processing, metabolism, and function [12]. The accumulated evidence suggests a critical role for m7G in human disease development, especially cancer, and aberrant expression of m7G is strongly associated with tumorigenesis and progression [13]. However, the detailed molecular mechanisms of m7G-related lncRNAs in treatment and prognosis of UCEC remain to be elucidated.

The past decade has witnessed remarkable advances in cancer immunotherapy, including chimeric antigen receptor T cells, bispecific antibodies, and immune checkpoint inhibitors [14,15,16,17]. Immunotherapy is a promising approach to treat advanced or recurrent UCEC patients. In the wake of immunotherapy and personalized medicine, it is necessary to identify biomarkers to predict treatment response.

The aberrant expression or mutation of lncRNAs and the abnormal modifications of m7G lead to diverse disorders that include many cancers. However, the direct interconnection and role of lncRNAs and m7G in EC remain largely unknown and require further investigation. In this study, we used the TCGA database to search for m7G-related lncRNAs. We ultimately identified six differentially expressed lncRNAs and successfully constructed an EC risk prediction model. In addition, the m7G-related lncRNAs model was used as the target to explore potential therapeutic drugs, in order to find new modes of immunotherapy.

## 2. Materials and Methods

### 2.1. Data Collection

**Figure 1:**
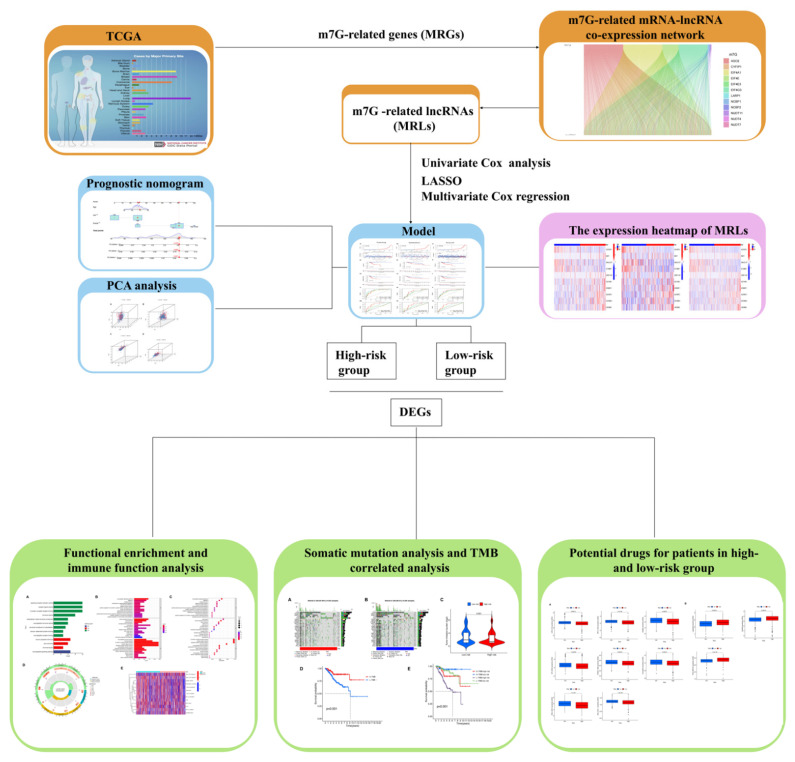
The workflow chart.

The workflow chart (Figure 1) shows the process of data preparation, data analysis, and visualization of the results in our study. The RNA-seq profiles, clinical data and information on somatic mutations of UCEC cases, and normal cases were all downloaded from the TCGA database [18]. A total of 20 m7G-related genes (MRGs) were obtained from a previous study.

### 2.2. Identification of m7G-Related lncRNAs in UCEC

The m7G-related mRNA-lncRNA co-expression network was identified utilizing “limma” package in R software. The correlation was considered significant, with a |Pearson correlation coefficient | > 0.4 and the *p*-value <0.05. The coexpression network is visualized in the Sankey diagram.

### 2.3. Construction of m7G-Related lncRNAs Prognostic Signature for UCEC

Cox univariate analysis was utilized to evaluate the prognostic values (*p* < 0.05) of m7G-related lncRNAs (MRLs), among which 15 MRLs remained after filtering. To avoid overfitting, the least absolute shrinkage and selection operator (LASSO) regression was then applied for further analysis. Finally, risk scores were obtained through the multivariate Cox regression. The risk scores for each patient were calculated using the formula: Risk score = Expression_lncRNA1_ × Coefficient _lncRNA1_ + Expression _lncRNA2_ × Coefficient _lncRNA2_ + Expression _lncRNAn_ × Coefficient _lncRNAn_ [19]. We separated a total of 541 patients into training or validation groups, randomly, with the ratio of 1:1. The clinicopathological characteristics of patients with UCEC are shown in Table 1. Patients in both the training and validation cohorts were divided into high-risk groups and low-risk groups, according to their risk scores. Overall survival (OS) and progression-free survival (PFS) were generated for all patients, train group, and validation group, respectively, using the “survminer” R package. In addition, the receiver operating characteristic curve (ROC) [20] and the area under the ROC curve (AUC) were constructed to identify the predictive accuracy of prognostic signatures utilizing the “timeROC” R package.

### 2.4. Construction of Predictive Nomogram and Principal Component Analysis

A prognostic nomogram was formulated via the “rma” package in R software to predict the probability of 1-, 3-, and 5-OS of UCEC patients. To evaluate the accuracy of the nomogram, calibration curves were then constructed. Principal component analysis (PCA) was performed to explore the distribution of patients with various risk scores utilizing the “scatterplot3D” R package.

### 2.5. Functional Enrichment Analysis and Immune Function Analysis

We explored the differentially expressed genes (DEGs) between low-risk and high-risk groups using the “limma” package with the threshold of FDR < 0.05 and |log_2_FC| ≥ 1. Gene Ontology (GO) [21] analysis was performed, based on the DEGs, to explore the enrichment of biological processes (BP), molecular functions (MF), and cellular components (CC). The immune function of DEGs was also identified, based on the “immune.gmt”, and visualized in a heatmap.

### 2.6. Somatic Mutation and Tumor Mutation Burden in Different Risk Groups

The cases were divided into low-risk and high-risk groups, according to their risk scores. The “maftools” [22] and “ggpubr” packages in R software were utilized to visualize the mutation data and tumor mutation burden (TMB), respectively, between different risk groups.

### 2.7. Drug Sensitivity Prediction between Different Risk Groups

We predicted the IC_50_ values of various chemotherapy drugs for the low-risk and high-risk UCEC groups via the “pRRophetic” [23] R package. The *p*-value indicates the effectiveness in inhibiting biochemical activity or biological processing.

### 2.8. Statistical Analysis

R version 4.1.1 was utilized to perform and visualize statistical analysis, while Pearson correlation analysis was used to identify the correlations among variables. Student’s *t*-test was performed for normally distributed variables between two groups, while the Mann–Whitney U-test was conducted for abnormally distributed variables.

## 3. Results

### 3.1. Identification of Prognostic m7G-Related lncRNAs in UCEC

**Figure 2:**
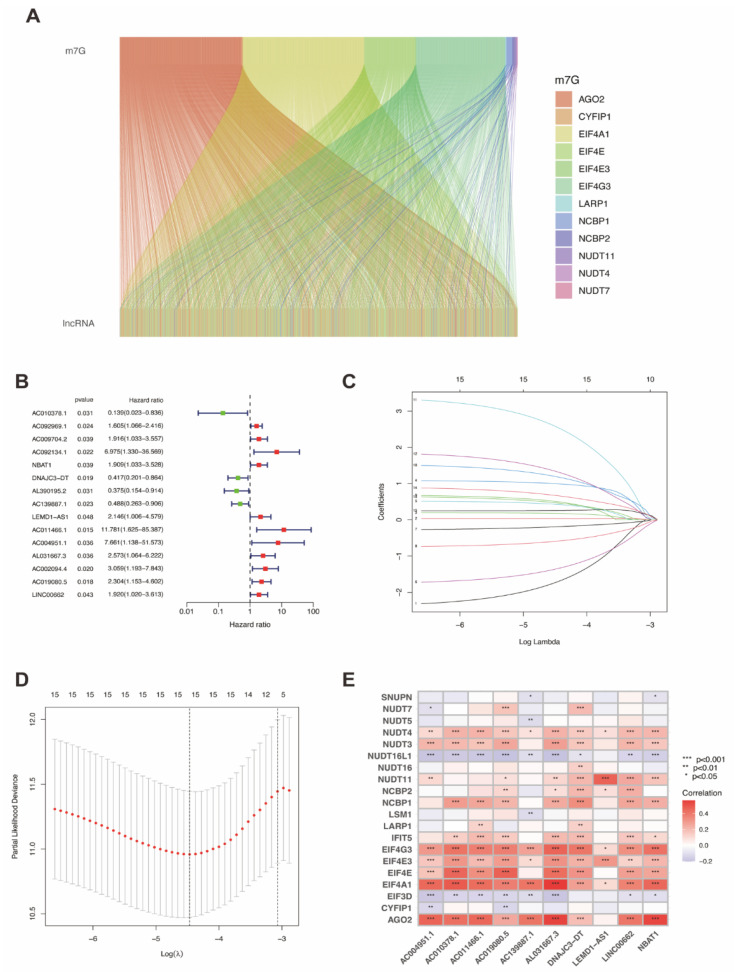
Identification of prognostic m7G-related lncRNAs. (**A**) Sankey diagram of the correlation between MRGs and lncRNAs. (**B**) Univariate Cox regression analysis identified 10 MRLs correlated with prognosis. (**C**,**D**) Cvfit and lambda curves of Lasso regression analysis. (**E**) The correlation between 19 MRGs and 6 MRLs identified by multivariate Cox regression.

We constructed the Sankey diagram (Figure 2A) to visualize the co-expression network of lncRNAs and MRGs, based on which, we obtained m7G-related lncRNAs. Then, we applied univariate Cox analysis for primary screening and 15 MRLs were extracted after filtering (Figure 2B). Lasso regression analysis was also applied, as shown in Figure 2C–D. Finally, the multivariate Cox regression analysis was utilized to identify 10 MRLs, including AC010378.1, NBAT1, DNAJC3-DT, AC139887.1, LEMD1-AS1, AC011466.1, AC004951.1, AL031667.3, AC019080.5, and LINC00662.

The relationship between the 10 MRLs and 20 MRGs was identified, as shown in Figure 2E. The color red represents a positive correlation between MRLs and MRGs, while blue indicates a negative correlation. For instance, AC004951.1 was significantly positively correlated with NUDT3, NUDT4, NUDT11, EIF4G3, EIF4E3, EIF4E, EIF4A1, and AGO2, but was significantly negatively correlated with NUDT7, NUDT16L1, EIF3D, and CYFIP1. Some MRGs had a significantly positive correlation with most or even all MRLs, such as NUDT3, NUDT4, NUDT11, NCBP2, NCBP1, IFIT5, EIF4G3, EIF4E3, EIF4E, EIF4A1, and AGO2, whereas some MRGs had a significantly negative correlation with multiple MRLs, including NUDT16L1 and EIF3D. LARP1 was significantly positively correlated with two MRLs, whereas SNUPN and CYFIP1 were significantly negatively correlated with two MRLs. Both NUDT5 and LSM1 had a significantly negative relationship with AC139887.1. NUDT7 was significantly positively correlated with AC019080.5 and DNAJC3-DT, but negatively related to AC004951.1.

**Figure 3:**
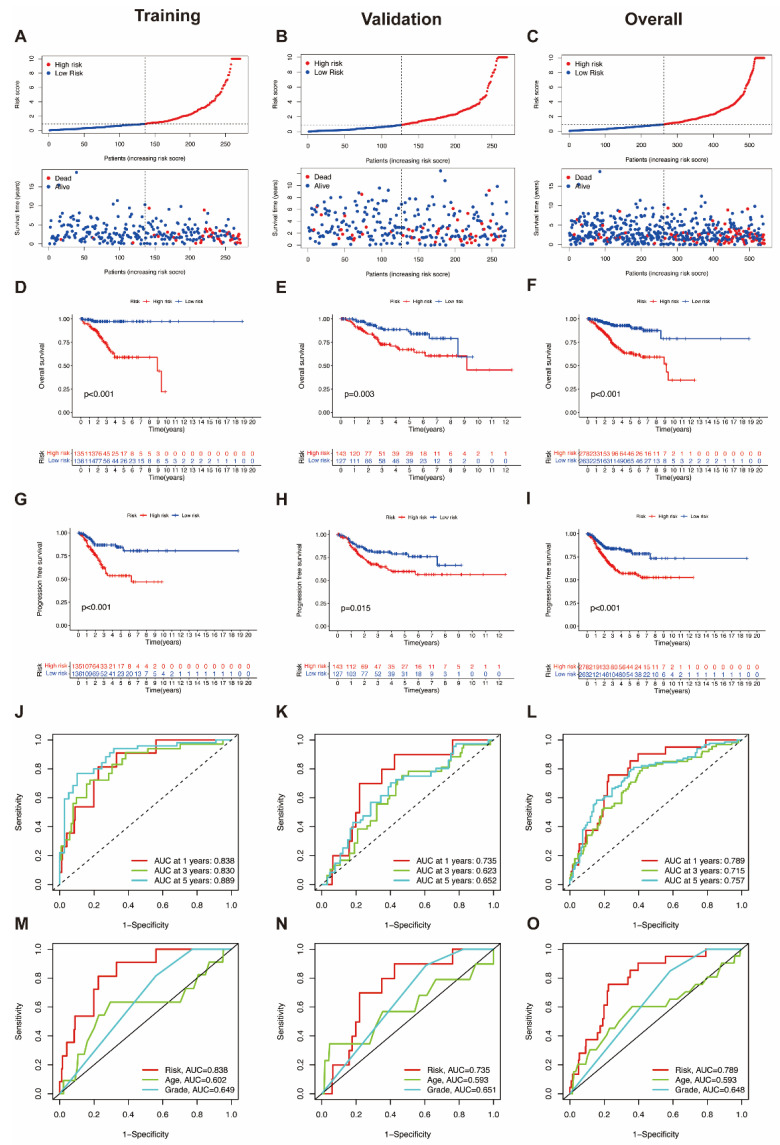
Construction and validation of the prognostic model based on MRLs. (**A**–**C**) The distribution of risk scores and overall survival status in the training group, validation group and overall group. (**D**–**F**) The Kaplan–Meier curves of overall survival rates in the training group, validation group and overall group. (**G**–**I**) The Kaplan–Meier curves of progression-free survival rates in the training group, validation group and overall group. (**J**–**L**) Time-dependent ROC curves for the training group, validation group, and overall group. (**M**–**O**) Clinicopathological characteristics ROC curves for the training group, validation group, and overall group.

Construction and validation of a prognostic model in UCEC based on MRLs:

To identify the prognostic value and predictive accuracy for endometrial cancer of these MRLs, a UCEC prognostic model for predicting risk scores was constructed, based on the 10 MRLs extracted, as above. To evaluate the ability of predicting prognostic value, the model was subsequently trained and validated. We identified the distribution of risk scores and overall survival status in the training group, which indicated that the samples in the low-risk and high-risk groups were distributed fairly (Figure 3A). The overall survival (OS) rate (Figure 3D) and the progression free survival (PFS) rate (Figure 3G) in the training group were generated via Kaplan–Meier survival analysis. Both the OS rate and PFS rate showed that the prognostic outcome of UCEC patients in the high-risk group was worse than that of the low-risk group. ROC curves were formulated to validate the predictive accuracy, including a time-dependent ROC (Figure 3J) and an ROC based on clinicopathological characteristics (Figure 3M). The same analyses were also applied in the validation group (Figure 3B,E,H,K,N) and overall group, respectively, (Figure 3C,F,I,L,O). The results in the validation group and overall group both illustrated that patients in the high-risk group could have worse survival situations and higher mortality rates than those in the low-risk group. In addition, the expression of the 10 MRLs in the low- and high-risk groups were also visualized, using data from the training group (Figure S1A), the validation group (Figure S1B), and the overall group (Figure S1C). Overexpression of an MRL in the high-risk group indicated it as a risk factor for UCEC, whereas upregulated MRLs in the low-risk group symbolized protective factors. These expression heatmaps further illustrated the predictive value of the 10 MRLs, consisting of 7 UCEC risk factors: NBAT1, LEMD1-AS1, AC011466.1, AC004951.1, AL031667.3, AC019080.5, and LINC00662; and 3 UCEC protective factors: AC010378.1, DNAJC3-DT, and AC139887.1.

### 3.2. Construction of a Predictive Nomogram, Identification of Independent Prognostic Factors and PCA Analysis

**Figure 4:**
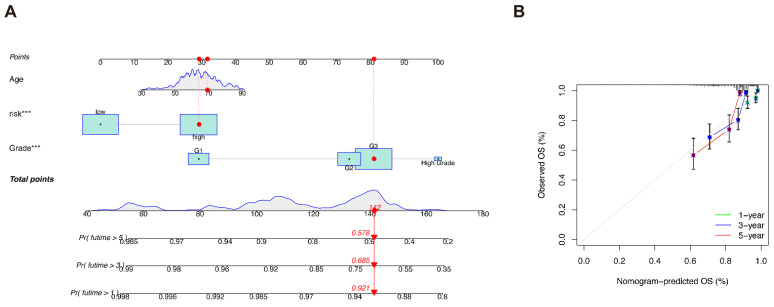
The predictive nomogram. (**A**) The predictive nomogram based on both clinicopathological characteristics and risk classifications. (**B**) Calibration curves of the nomogram. *** *p* < 0.001.

We plotted a predictive nomogram based on both clinicopathological characteristics and risk group classification (Figure 4A), and constructed calibration curves to illustrate the prediction accuracy of the nomogram (Figure 4B).

In addition, we also identified the independent prognostic factors for UCEC patients. The results of univariate and multivariate Cox regressions are shown in Figures S2A,B, respectively. The concordance index (Figure S2C) proves that the results of the predictive model are believable.

**Figure 5:**
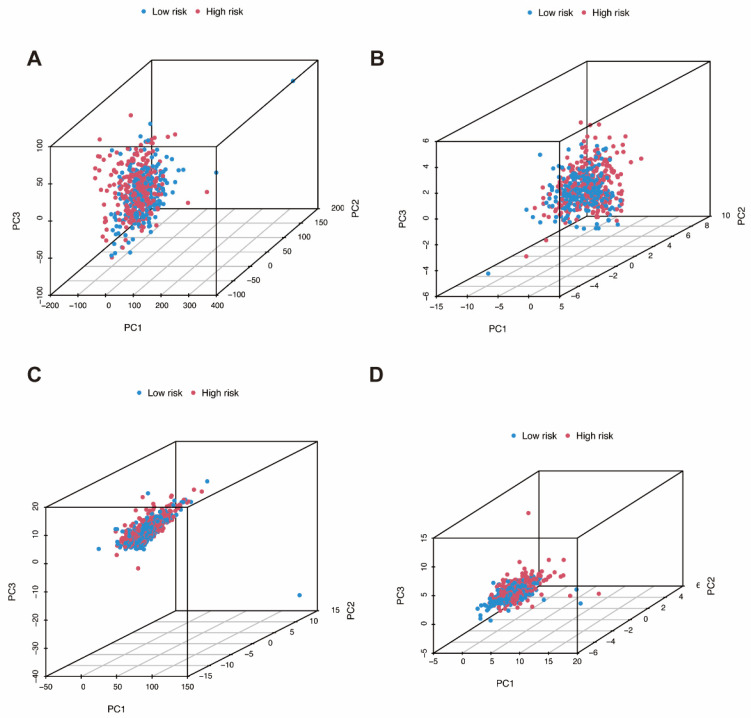
Principal component analysis. (**A**) The distribution of all genes. (**B**) The distribution of m7G-related genes. (**C**) The distribution of m7G-related lncRNAs. (**D**) The distribution of the m7G-related lncRNAs prognostic signature.

We applied principal component analysis (PCA) to identify the distribution of samples in low-risk and high-risk groups. The distributions of all genes, m7G-related genes, m7G-related lncRNAs, and the m7G-related lncRNAs prognostic signature are shown in the 3D PCA maps, respectively (Figure 5A–D).

### 3.3. Functional Enrichment and Immune Function Analysis

**Figure 6:**
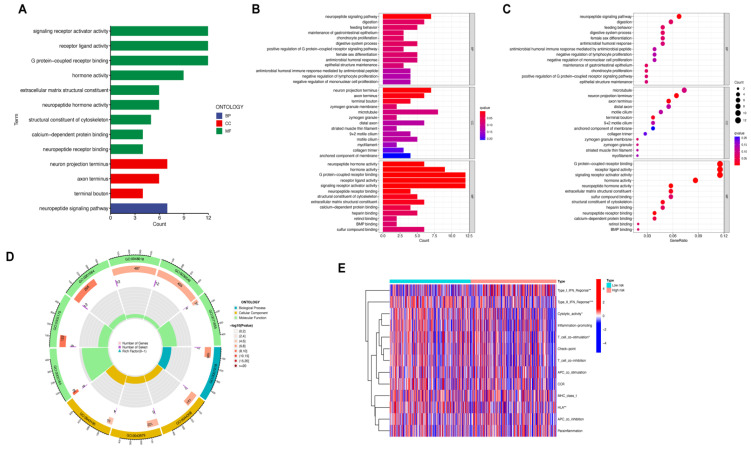
Functional enrichment and immune function analysis. (**A**–**D**) Gene ontology enrichment analysis of DEGs. (**E**) Immune function analysis of DEGs.

After establishing the prognostic model based on MRLs for UCEC, we identified a low-risk group and a high-risk group. Differentially expressed genes were then extracted from these two different risk groups (Table S1, shown in Figure S1D–E), and were analyzed using GO and immune function analysis. The DEGs were obviously enriched into biological processes (BP), cellular component (CC), and molecular function (MF) in GO analysis (Table S2). The DEGs were mainly enriched in MF, consisting of: G-protein-coupled receptor binding, receptor ligand activity, signaling receptor activator activity, hormone activity, neuropeptide hormone activity, extracellular matrix structural constituent, structural constituent of cytoskeleton, calcium-dependent protein binding, and neuropeptide receptor binding. The DEGs were significantly involved in the neuropeptide signaling pathway, in terms of BP, while DEGs were mainly enriched in the neuron projection terminus, axon terminus, and terminal bouton, in terms of CCs. G-protein-coupled receptors (GPCRs) are the largest family of cell-surface molecules involved in signal transmission. Available studies have demonstrated that GPCRs have recently emerged as crucial players in tumor growth and metastasis [24,25]. The role of pituitary gonadotropin-releasing hormone receptors (GnRH-R), involved in G-protein-coupled receptors (GPCRs) mediated intracellular signaling pathway, are proven to be crucial in various cancers, such as endometrial cancer [26]. Growing evidence supports the importance of receptor ligand activity in tumor responses to prognosis and therapy. Receptor ligand activity has recently been designated as an especially useful clinical target [27]. A calcium-dependent phospholipase A2 (cPLA2), related to calcium-dependent protein binding, was identified as a novel target and played an essential role in endometrial tumorigenesis [28]. Various neuropeptides, consisting of hypothalamic decapeptide GnRH, neuropeptide Y, and leptin were proven to have a tight association with endometrial cancer [29,30,31]. The above research findings proved that those pathways are potentially correlated with the mechanism of UCEC and our GO analysis results are credible. All the results of GO analysis are shown in Figure 6A–D.

Immune function analysis was also applied to explore the potential immune signatures correlated with the pathological and molecular mechanisms of UCEC (Figure 6E). The significantly differential immune function between low-risk and high-risk groups consisted of type I IFN response, type II IFN response, human leukocyte antigen (HLA), T cell co-stimulation, and cytolytic activity.

### 3.4. Somatic Mutation, TMB Correlated Analysis and Drug Sensitive Prediction in UCEC

**Figure 7:**
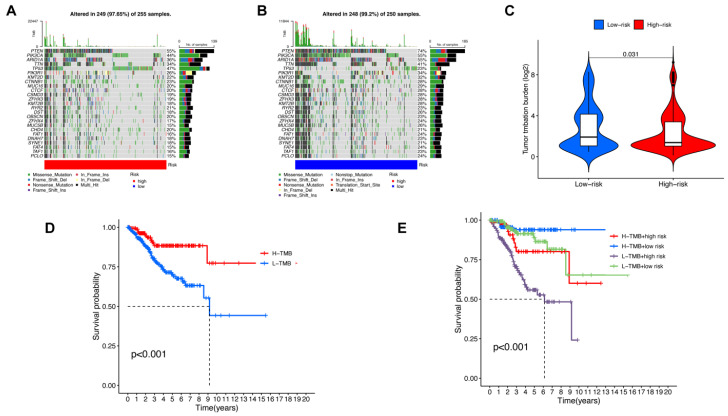
Somatic mutation analysis and TMB correlated analysis. (**A**) The waterfall chart of the top 25 most frequently mutated genes in the high-risk group. (**B**) The waterfall chart of the top 25 most frequently mutated genes in the low-risk group. (**C**) TMB in low-risk and high-risk groups. (**D**) Kaplan–Meier curves of OS in high-TMB and low-TMB groups. (**E**) Kaplan–Meier curves of OS in groups with various TMB and risk scores.

Based on DEGs extracted previously, we then explored genomic differences in somatic mutation between low-risk and high-risk groups. The results of somatic mutation analysis are presented in Figure 7A,B, which shows that phosphatase and tensin homolog (PTEN), phosphatidylinositol-4,5-bisphosphate 3-kinase catalytic subunit alpha (PIK3CA), AT-rich interaction domain 1A (ARID1A), titin (TTN), and tumor protein p53 (TP53) were most frequently mutated in the high-risk group, while PTEN, PIK3CA, ARID1A, TTN, and phosphoinositide-3-kinase regulatory subunit 1(PIK3R1) were mainly mutated in low-risk group. It also shows that TTN, PTEN, PIK3CA, and ARID1A were the most frequent mutated genes, not only in the high-risk group, but also in the low-risk group. Established evidence proves that co-mutations in PIK3R1 and PIK3R2, the members of PI3K-Akt signaling pathway, are mainly involved in the development of endometrial cancer [32]. In addition, the lower mutation rates of TTN, PTEN, PIK3CA and ARID1A have been identified as possible risk factors in endometrial cancer [33]. ARID1A mutation also plays an important role in the activation of the PI3K/AKT/mTOR pathway [34].

The tumor mutation burden (TMB) varied from the high-risk group to the low-risk group, showing that TMB is significantly lower in the high-risk group (Figure 7C). Kaplan–Meier analysis was performed to identify the correlation between TMB in UCEC and survival probability (Figure 7D). The results indicated that patients with high TMB have a better prognosis. The relationship among TMB, risk scores based on MRLs, and prognosis was subsequently identified, showing that the prognosis of patients with high risk scores and low TMB is the worst.

**Figure 8:**
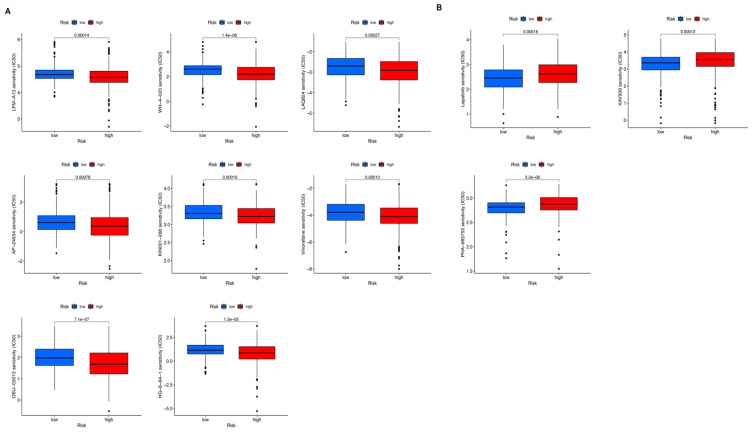
Potential drugs for endometrial cancer patients. (**A**) Potential drugs for patients in the high-risk group. (**B**) Potential drugs for patients in the low-risk group.

Drug sensitive prediction analysis was applied, based on DEGs, to explore potential drugs for UCEC patients. The landscape of 11 candidate drugs are shown in Figure 8. The drugs in Figure 8A could be ideal candidate drugs (LFM-A13, WH-4-023, LAQ824, AP-24534, KIN001-266, Vinorelbine, OSU-03012, and HG-6-64-1) for treating patients in the high-risk group, while drugs in Figure 8B might be potential drugs (Lapatinib, XAV939 and PHA-665752) for patients in the low-risk group.

## 4. Discussion

Although the cornerstone of management for UCEC patients is surgery, the prognostic difference between various types of endometrial cancers is substantial [35]. Hence, it is of great significance to identify the prognosis of endometrial cancers with molecular heterogeneity. The distribution of m7G exists widely in the human body [36]. It is known that lncRNA acts as a therapeutic target and innovative biomarker for a variety of cancers, because of its tissue-specific expression characteristics and genome-wide expression patterns [37]. However, studies about the prognostic model based on m7G-related lncRNAs for UCEC patients are still limited.

In our study, we identified 10 m7G-related lncRNA signatures to predict prognosis for UCEC patients. UCEC patients were randomly distributed into a training group and a validation group. The training group was used for constructing the prognosis model, while the validation group was for testing the reliability of the model. Each patient had an individual risk score, according to which, patients were assigned to high-risk or low-risk groups. The prognostic outcome between high-risk and low-risk groups was significantly different. The prediction ability was identified and visualized in ROC curves. The predictive nomogram, taking account of both clinicopathological characteristics and risk scores, was established, and PCA analysis was applied to intuitively illustrate the distribution of patients with various risk scores.

Among the 10 MRLs, NBAT1, LEMD1-AS1, AC011466.1, AC004951.1, AL031667.3, AC019080.5, and LINC00662 had a higher expression in the high-risk group, whereas AC010378.1, DNAJC3-DT, and AC139887.1 were overexpressed in the low-risk group. It is known that NBAT1 plays an essential role in suppressing malignant cell proliferation and migration in multiple kinds of cancers, including renal carcinoma [38], hepatocellular carcinoma [39], and glioma [13]. LEMD1-AS1 is reported to act as a prognostic signature for ovarian cancer [40], while AL031667.3 and AC019080.5 are proven to be capable of predicting the outcome of lung adenocarcinoma [41]. LINC00662 is shown to promote the development and progression of cancer and have relationships with a wide range of tumors in various systems [42], such as the reproductive, respiratory, and nervous systems. However, the functional mechanism of the other MRLs have rarely been studied and remain unknown. These lncRNAs might influence the pathogenesis and development of UCEC through becoming involved in N^7^-methylguanosine regulation. Further studies should be performed to reveal the underlying mechanism of the relationship between MRLs and UCEC.

The differentially expressed genes between the high- and low-risk groups were identified and utilized for functional enrichment and immune function analysis. Type I interferon (IFN) is known for its ability to influence immune response and activate antiviral program [43]. Type I IFNs can be secreted by intratumoral dendritic cells or malignant cells, and then lead to anticancer effects, consisting of promoting terminal differentiation and inhibiting cell cycle progression [44]. Type II IFNs play an essential role in regulating both adaptive and innate immune responses and preventing the development and progression of tumors [45]. In our study, Type I IFN and Type II IFN were proven to be significantly correlated with endometrial cancer, indicating that targeting type I and II IFN into a special cellular compartment of endometrial cancer might manage to play an optimal therapeutic role. The human leukocyte antigen has the ability of distinguishing between non-self and self-peptides, which enables HLA to play a key role in activating a host immune response against tumor cells and pathogens [46]. T cell co-stimulation can contribute towards reprogramming various immune regulatory pathways and enhance counter-tumor immunity [47]. Cytolytic activity plays a crucial role in mutation. Tumors with higher cytolytic activity were associated with more mutations [48] and this conclusion was consistent with our findings that the low-risk group showed higher cytolytic activity as well as higher somatic mutations. In consideration of the close relationship between cytolytic activity and anti-regulatory immune responses, the mutations correlated with cytolytic activity could act as novel biomarkers for predicting prognostic outcomes and exploring potential immune treatments. HLA, T cell co-stimulation and cytolytic activity were all identified as being significantly correlated with endometrial cancer, in our study. Further study about endometrial cancer should be applied to obtain a better understanding of its underlying immune mechanism, which could help to promote the development of potential tumor immunotherapy.

In recent years, the essential role that lncRNA plays in mediating tumor progression and regulating therapy resistance in a variety of cancers has been elucidated more and more clearly [49]. N^7^-methylguanosine is a novel kind of internal modification that is widespread in human mRNA. Hence, there are numerous unexplored areas remaining between m7G and lncRNAs. We tried to provide a potential target for tumor therapy and future research of endometrial cancer, but, inevitably, there were some limitations in our study. First, only the TCGA database was utilized for constructing and validating the model. To improve the reliability of this MRLs model, more additional samples could be included. Second, it is better to validate the expression of MRLs and DEGs through other methods or experiments. Finally, it remains unknown how those lncRNAs interact with N^7^-methylguanosine and how the underlying biological mechanism of m7G-related lncRNAs involved in endometrial cancer works. Further studies should be performed to clarify these scientific issues.

## 5. Conclusions

In conclusion, we constructed a model based on 10 MRLs for prognostic prediction. Compared with previous clinicopathological methods, the advantage of this model lies in its convenience for testing in patients. The findings in our study might help to provide novel insights into predicting the prognosis of endometrial cancer patients and assist in exploring the underlying mechanism of m7G-related lncRNAs’ interaction with endometrial cancer.

## Figures and Tables

**Table 1:** Clinicopathological characteristics of patients with UCEC.

Characteristics		Total		Train		Validation		*p*-Value
		n	%	n	%	n	%	
Age (years)	≤65	304	56.19%	152	56.09	152	56.3	0.9521
	>65	235	43.44%	119	43.91	116	42.96	
	unknow	2	0.37%	0	0	2	0.74	
Grade	G1	98	18.11%	55	20.3	43	15.93	0.528
	G2	120	22.18%	61	22.51	59	21.85	
	G3	312	57.67%	149	54.98	163	60.37	
	High Grade	11	2.03%	6	2.21	5	1.85	

## Data Availability

The datasets for this study can be found in the TCGA database. Please see the https://portal.gdc.cancer.gov for more details.

## Supplementary Materials

The following supporting information can be downloaded at: https://www.mdpi.com/article/10.3390/genes13081301/s1. Figure S1. The expression heatmap of MRLs and DEGs; Figure S2. Independent prognostic factors for UCEC patients; Table S1. The differentially expressed genes (DEGs) between low-risk and high-risk groups; Table S2. The enriched GO terms of differentially expressed genes.
