# Supplementary material for: Identification and Validation of an m7G-Related lncRNAs Signature for Prognostic Prediction and Immune Function Analysis in Endometrial Cancer
Source: Genes (Basel). 2022 Jul 22;13(8):1301. doi: 10.3390/genes13081301 (PMC9330151; doi:10.3390/genes13081301)
Supplement: Supplementary file 1 [file genes-13-01301-s001.zip › SUPPLEMENTARY INFORMATION.pdf]

SUPPLEMENTARY INFORMATION

Identification and Validation of an m7G-Related lncRNAs Signature for Prognostic Prediction and Immune Function Analysis in Endometrial Cancer

Jiani Sun †, Li Li †, Hong Chen, Lei Gan, Xiaoqing Guo and Jing Sun *

Department of Gynecology, Shanghai First Maternity and Infant Hospital, School of Medicine, Tongji University, Shanghai 200092, China; sunjiani5947@163.com (J.S.); 1931199@tongji.edu.cn (L.L.); chenhong377@163.com (H.C.); gan_leilei@126.com (L.G.); xiaoqing_guo@tongji.edu.cn (X.G.)

* Correspondence: sunjing61867@tongji.edu.cn

† These authors contributed equally to this work.

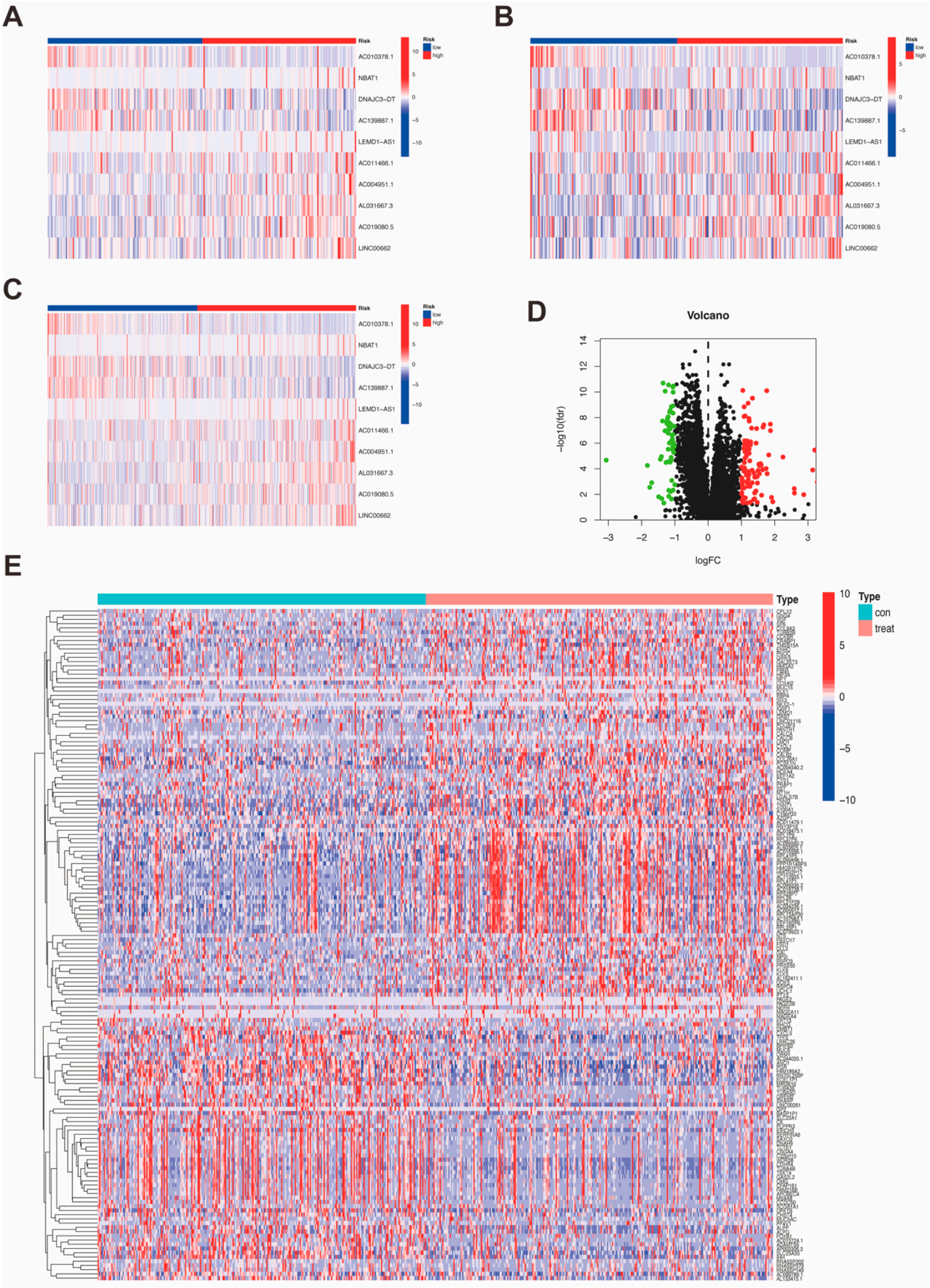

Figure S1. The expression heatmap of MRLs and DEGs. (A-C) The expression heatmap of 6 MRLs in the training group, validation group and overall group. (D) The expression volcano plot of DEGs. (E) The expression heatmap of DEGs

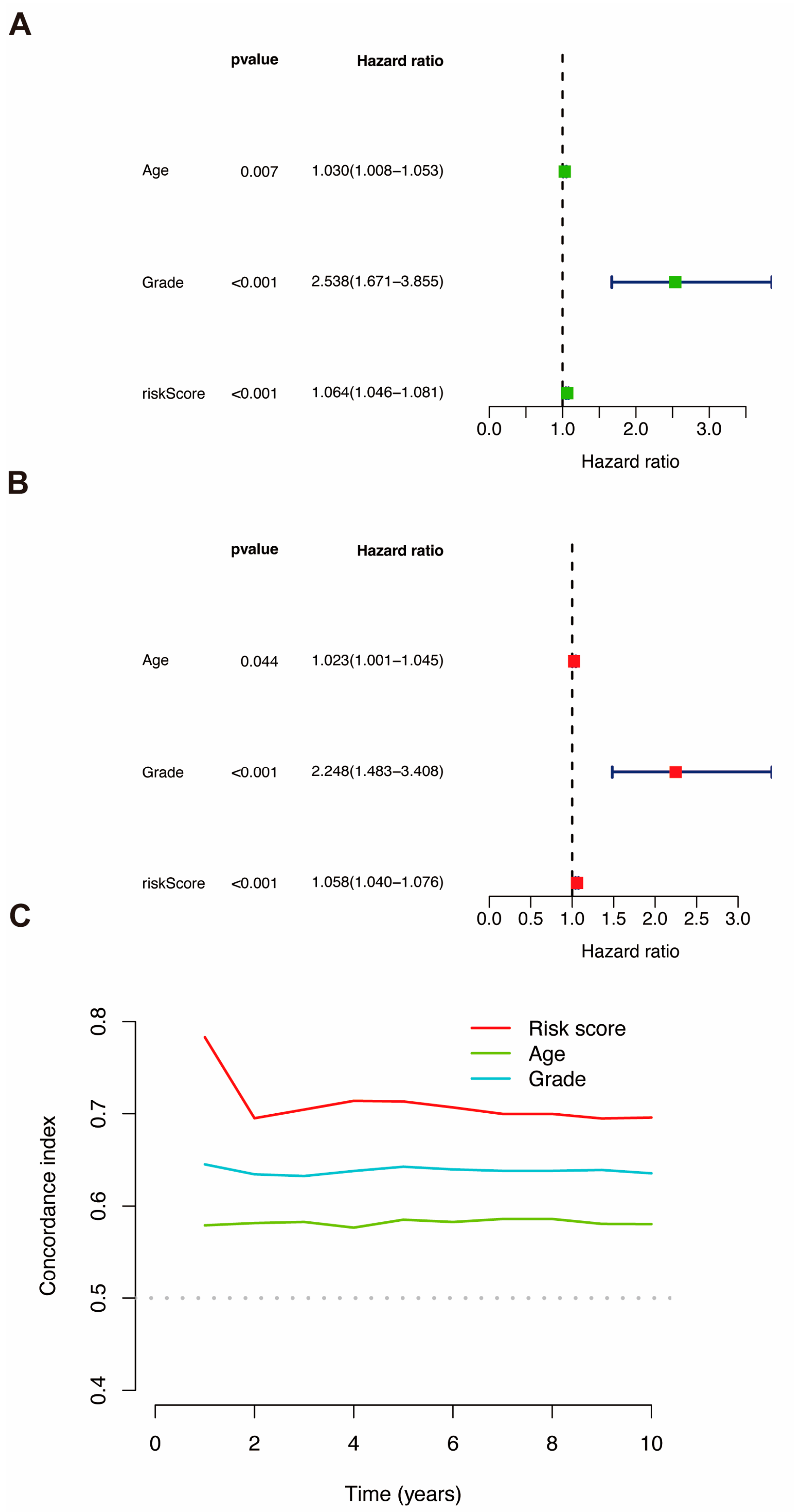

Figure S2. Independent prognostic factors for UCEC patients. (A) Independent prognostic factors identified by univariate Cox regression. (B) Independent prognostic factors identified by multivariate Cox regression. (C) The concordance index of the model.
