# Supplementary material for: Identification and Validation of an m7G-Related lncRNAs Signature for Prognostic Prediction and Immune Function Analysis in Endometrial Cancer
Source: Genes (Basel). 2022 Jul 22;13(8):1301. doi: 10.3390/genes13081301 (PMC9330151; doi:10.3390/genes13081301)
Supplement: Supplementary file 1 [file genes-13-01301-s001.zip › SUPPLEMENTARY TABLE.pdf]

SUPPLEMENTARY TABLE

Identification and Validation of an m7G-Related lncRNAs Signature for Prognostic Prediction and Immune Function Analysis in Endometrial Cancer

Jiani Sun †, Li Li †, Hong Chen, Lei Gan, Xiaoqing Guo and Jing Sun *

Department of Gynecology, Shanghai First Maternity and Infant Hospital, School of Medicine, Tongji University, Shanghai 200092, China; sunjiani5947@163.com (J.S.); 1931199@tongji.edu.cn (L.L.); chenhong377@163.com (H.C.); gan_leilei@126.com (L.G.); xiaoqing_guo@tongji.edu.cn (X.G.)

* Correspondence: sunjing61867@tongji.edu.cn

† These authors contributed equally to this work.

Table S1 The differentially expressed genes (DEGs) between low-risk and high-risk groups

Gene Symbol	LowMean	HighMean	logFC	P.Value	FDR
NKX2-1	0.701069	2.69425709	1.94225917	0.01700992	0.03796066
DMBT1	6.58762328	2.43656781	-1.4349077	1.47E-06	1.81E-05
AC005912.1	12.2055487	28.5586696	1.22639162	1.49E-10	1.16E-08
AC090498.1	10.9575867	26.4391318	1.27074471	5.79E-08	1.35E-06
PTX3	0.80353958	1.86677278	1.21610534	4.99E-05	0.00032014
OMG	5.11519343	2.14466203	-1.2540385	2.82E-10	2.06E-08
KLK5	2.59822474	6.83328758	1.39505362	0.00888821	0.02238887
RNA5SP370	2.59507338	0.92541823	-1.4875979	0.0056055	0.01541161
HIF3A	0.86917294	1.98168383	1.18901165	5.99E-08	1.38E-06
FBXO17	1.5291965	3.43364664	1.16696777	3.32E-05	0.00022593
C1QL1	2.56263695	6.76622522	1.4007221	7.68E-05	0.00045799
MS4A8	19.6219184	8.5170115	-1.2040469	2.51E-06	2.77E-05
RSPO3	1.51822378	3.91706602	1.36738899	0.01008544	0.02478292
COL9A3	2.4282941	5.23658567	1.1086833	1.14E-06	1.45E-05
AC018475.1	0.76115269	1.52697193	1.00441575	8.55E-06	7.39E-05
GAL3ST3	0.90868474	2.05591518	1.17792898	0.00029644	0.00138591
UPK1B	37.7700554	18.0682091	-1.0637894	8.89E-08	1.90E-06
CXCL5	10.3932678	5.19625779	-1.0001044	0.00039719	0.001756
GRIK5	1.33246866	2.71790156	1.0283916	0.00080046	0.00316419
AC244035.1	2.56243781	1.23572417	-1.0521602	0.00127615	0.00465539
ALPP	21.4802681	9.48303357	-1.1795915	3.41E-10	2.44E-08
CYS1	2.68590004	6.42191165	1.25759721	0.00057088	0.00239282
TFF3	833.832882	353.892452	-1.2364473	4.41E-10	2.96E-08
RPL41P5	25.5698087	54.0831117	1.08073679	1.33E-07	2.52E-06
COL26A1	8.18490028	16.7732072	1.03512183	0.00058143	0.00242641
AC107983.1	1.84514983	5.56878928	1.59362573	5.61E-06	5.24E-05
RPL13AP20	2.59737961	6.10503949	1.23294375	2.51E-10	1.87E-08
SST	19.1713732	115.61877	2.59235004	0.00247806	0.00795691
SCGB1A1	28.3108392	12.4630888	-1.1836929	0.0013497	0.00488499
S100A1	9.82836273	21.8543901	1.15290011	1.79E-07	3.18E-06
CALB2	1.14270719	10.0727146	3.13992487	1.63E-05	0.00012501
POU3F3	1.5708231	3.30742662	1.07418843	2.12E-05	0.0001553
SLC23A1	3.02113245	1.36734569	-1.1437114	5.48E-10	3.44E-08
ORM1	15.7579175	5.99590228	-1.3940281	0.00847932	0.02154132
PAGE2	1.14388853	4.27648493	1.90247898	0.00646878	0.01729643
PPP1R14BP3	16.538566	34.030963	1.04101383	2.23E-13	7.48E-11
UCHL1	20.0102719	45.9848857	1.20041898	4.36E-12	7.52E-10
LRRC26	6.87108733	3.06488815	-1.164704	8.83E-10	4.90E-08
EEF1B2P6	0.63062936	1.71045986	1.43952001	3.99E-08	9.89E-07
FSD1	0.68535822	1.71058134	1.31955655	1.70E-05	0.00012993

COX4I2	1.34935093	4.93013362	1.86936114	5.02E-10	3.25E-08
RSPO4	0.92421563	2.39716931	1.37503042	3.27E-05	0.00022258
AP003306.2	1.80709346	0.8704608	-1.0538199	6.05E-09	2.16E-07
AC099560.2	0.86989736	1.94767277	1.16283421	2.28E-07	3.87E-06
KLK6	9.41544675	19.4572235	1.0472044	0.02148312	0.04581003
PGR	15.2907759	7.4147702	-1.0441877	7.73E-14	3.34E-11
TEKT4	3.82877832	1.6662221	-1.2003034	1.63E-07	2.97E-06
AC016596.1	4.29785078	13.3798542	1.6383751	1.51E-05	0.00011786
AC004540.2	1.94682082	4.384312	1.17123036	2.33E-08	6.58E-07
APOBEC4	2.01059792	0.87494445	-1.2003613	4.26E-09	1.64E-07
FBN3	1.14438956	2.29784792	1.00570507	0.0030218	0.00937603
MUC5AC	3.16524616	1.45195312	-1.1243228	0.00174757	0.00602516
CRABP1	12.7748486	37.150826	1.54008809	6.50E-06	5.90E-05
KRT13	5.86778803	2.33384102	-1.3301105	0.02266185	0.04779898
LMO1	0.60672615	1.49851809	1.30441909	1.30E-09	6.65E-08
FOXB1	1.74284062	0.80643772	-1.1118056	9.92E-07	1.29E-05
TNNT1	9.16300497	25.1378031	1.45596586	1.44E-09	7.19E-08
PRAP1	2.17046503	4.98653514	1.20003354	0.00055802	0.00234842
RPL37P6	1.24085065	2.66765642	1.10424338	2.89E-07	4.65E-06
GP2	5.10060091	0.61055312	-3.0624785	1.80E-06	2.13E-05
ECEL1P1	2.25067058	0.91948619	-1.2914551	2.71E-13	8.64E-11
ENHO	1.12508128	2.63883105	1.22986976	1.57E-07	2.89E-06
GAS2L2	3.77349739	1.77076611	-1.0915286	6.55E-09	2.30E-07
MIR3616	2.06629121	0.90508415	-1.1909198	5.51E-14	2.78E-11
RPL35P1	0.79930005	2.01153836	1.33149016	1.29E-12	3.03E-10
AC013724.1	4.27043882	1.88740107	-1.1779833	9.20E-10	5.04E-08
DLK1	0.27928504	2.71245256	3.2797877	0.00022375	0.00110841
NTS	6.74764911	49.3895019	2.87174756	0.00349434	0.01054492
CFAP161	3.3751271	1.50574971	-1.1644598	3.91E-08	9.70E-07
RPS28P7	144.99401	391.92398	1.43458055	0.00155973	0.00548706
AL603825.1	1.07614877	2.28468108	1.08611526	3.14E-06	3.31E-05
DNAH9	1.69054111	0.72583021	-1.2197811	1.44E-10	1.14E-08
NPW	2.68741215	6.0560753	1.17216555	0.00021712	0.00108264
SERPINA6	11.2683662	5.05285629	-1.1571073	3.25E-11	3.58E-09
C6	1.46991499	0.68107332	-1.1098507	1.73E-06	2.05E-05
RNA5SP202	18.1422541	0.71585228	-4.663548	0.01053421	0.02562664
MUC15	0.61754206	1.43275624	1.21418387	5.13E-05	0.00032705
ANO1	27.5004761	13.1893268	-1.0600857	1.07E-13	4.05E-11
EEF1A2	13.6038971	27.791431	1.03062012	0.0143229	0.03300965
NEFH	1.9037535	3.83613405	1.01080645	0.0024132	0.00779699
NPY	0.71220083	1.64883226	1.21108862	0.00041376	0.00181692
RBP4	3.52256571	8.50036037	1.27089739	0.01893758	0.04138248
MAGEA11	0.52903154	1.46370502	1.4681992	7.02E-05	0.00042387

LINC00261	8.46801436	4.121479	-1.0388616	3.87E-08	9.66E-07
LEMD1	2.20558787	4.56797231	1.05039067	0.01480023	0.03389606
SRARP	3.14929897	1.42781171	-1.141225	2.37E-11	2.81E-09
DLL3	0.37607567	1.78636436	2.24793149	9.23E-07	1.21E-05
IGSF1	0.21461052	1.97510267	3.20213493	1.99E-07	3.47E-06
BPIFB2	7.00783038	2.07572919	-1.7553496	0.00070043	0.00283456
CALCB	1.28123361	3.62086137	1.4987994	1.85E-05	0.00013848
AL162411.1	0.71798917	1.68393965	1.22980645	4.00E-07	6.09E-06
ADAMTS6	1.87687311	0.86915204	-1.1106506	2.55E-08	7.07E-07
CDHR4	9.46022562	4.18098675	-1.1780311	1.81E-09	8.41E-08
AC018738.1	7.90687149	28.976023	1.87368071	2.33E-09	1.04E-07
DOK5	0.8520314	1.94847371	1.19336596	2.21E-05	0.00016079
MT1H	3.69304049	9.24467747	1.32381391	0.00025578	0.00123213
WDR38	16.6305168	7.89427742	-1.0749539	1.64E-08	5.00E-07
AZGP1	1.55988703	4.33865622	1.47580673	0.0194706	0.04232413
MAGEA4	1.10542499	3.3642493	1.60568349	0.00397343	0.01167618
RPL21P28	1.38646248	3.24133283	1.2251786	3.82E-08	9.59E-07
GREM2	5.5532608	2.6025497	-1.0934094	0.00015402	0.00081331
RPL39	23.7485898	50.1375881	1.07805075	9.04E-11	8.15E-09
HOXA4	0.72653295	1.74643537	1.26531311	3.19E-08	8.44E-07
C22orf15	2.3313738	1.15168095	-1.0174392	8.09E-08	1.77E-06
AC113935.1	7.22660674	15.4180858	1.09323337	5.76E-08	1.35E-06
PLPPR3	3.37045157	1.40392438	-1.2634767	3.02E-06	3.21E-05
MUC6	15.255163	6.90280304	-1.1440434	2.80E-06	3.03E-05
CCKBR	0.78567513	1.58909508	1.01620066	0.00016382	0.00085706
CNGA4	2.92651884	1.14410901	-1.3549611	2.74E-14	2.00E-11
CALCA	5.5680687	11.599234	1.05878062	0.00647865	0.01730795
TUBA3E	1.84250556	0.51953246	-1.8263832	5.93E-06	5.49E-05
AL133415.1	1.82616131	0.84914036	-1.1047393	9.93E-12	1.37E-09
RN7SL250P	1.54685263	0.7457239	-1.0526223	3.48E-13	1.03E-10
ERICH3	2.03833016	0.93372015	-1.1263256	4.89E-10	3.22E-08
GAL	1.59581855	3.54348099	1.15087069	7.05E-07	9.67E-06
SP6	1.6138426	3.24982506	1.00986218	0.00228441	0.00747013
HMGN2P17	0.69965737	1.69460256	1.27622645	2.90E-08	7.85E-07
HMGB1P10	1.13725805	2.43068588	1.09580382	2.26E-08	6.41E-07
ALPG	66.6750304	32.5351577	-1.035147	3.25E-07	5.15E-06
SIX3	1.07725732	2.18992412	1.02351797	0.01127554	0.0271014
PRSS50	1.13737186	3.17188267	1.47963538	0.00027547	0.00130655
CHST4	1.5196834	0.63190646	-1.2659879	2.63E-09	1.12E-07
RNY3P16	0.69262372	1.47630924	1.09185126	1.04E-05	8.65E-05
TUBA3D	4.20358932	1.59058718	-1.4020623	8.35E-07	1.11E-05
AC079922.1	1.32355997	4.48364242	1.76024767	2.42E-13	7.90E-11
TUBA4B	11.6937176	5.30307388	-1.1408329	1.51E-09	7.47E-08

AC099336.2	2.61670826	8.32612407	1.66989197	6.07E-10	3.72E-08
FAM189A2	6.74020738	3.2400499	-1.0567769	4.27E-11	4.41E-09
RPL7P9	8.7930049	18.9774218	1.10985583	8.08E-11	7.44E-09
TNNI3	3.09778565	6.86730928	1.14850762	4.84E-05	0.0003122
HMGA2	1.41432557	2.85047738	1.0110893	8.90E-05	0.0005148
C19orf33	37.8522632	90.0825732	1.25086848	2.10E-06	2.40E-05
PNOC	1.92766345	5.74784045	1.57616682	3.96E-06	3.98E-05
GNG4	1.19940469	3.48566545	1.53911559	3.68E-05	0.00024638
VGF	1.05845834	3.74245607	1.8220209	5.78E-07	8.27E-06
PF4V1	1.52389481	0.70971646	-1.1024486	0.00912899	0.02286567
AC011479.1	0.98940567	2.0128469	1.02460337	0.0003754	0.00167755
TUBB2B	4.89652481	12.9011513	1.39766972	0.00239347	0.00774732
AC099789.1	5.43535877	1.67868477	-1.6950439	0.0002521	0.00122226
GRB7	14.139767	29.8843174	1.07963024	0.00078939	0.00313239
RPL41P1	41.8907649	131.111711	1.64609242	1.04E-09	5.58E-08
COMP	4.92976756	16.8412135	1.77240457	3.59E-05	0.00024159
FAM216B	6.38100309	2.5946302	-1.2982543	2.43E-09	1.07E-07
RHCG	1.54037083	3.48325766	1.17715948	0.02223477	0.04710945
TMSB15A	4.17177632	9.63073132	1.20698355	0.00012488	0.00068205
CCDC60	1.62224239	0.77244509	-1.0704851	1.88E-07	3.30E-06
IHH	42.2520964	20.2676886	-1.0598414	8.65E-12	1.24E-09
LGALS7B	1.85459854	5.33871257	1.52538495	4.43E-06	4.35E-05
BASP1P1	3.03269731	1.33754234	-1.181017	6.40E-10	3.87E-08
RNA5SP149	4.50155377	1.98248125	-1.1831158	0.00506877	0.01420671
AC034236.1	8.43740106	17.8631172	1.08211327	1.08E-11	1.44E-09
TCTE1	1.73445241	0.67017459	-1.3718714	2.45E-10	1.84E-08
SAXO2	1.86437269	0.91989439	-1.0191501	8.49E-10	4.76E-08
PANTR1	1.44164335	4.82223342	1.74198719	1.19E-05	9.64E-05
LINC01116	1.50887281	3.52351137	1.22354267	3.95E-05	0.00026199
PAGE2B	0.30416705	1.82672791	2.58632599	0.00092132	0.00355494
PCSK1N	13.6326421	28.1710794	1.04714965	0.00017533	0.00090887
CPLX2	1.02777225	2.23287034	1.11937887	0.00725605	0.01902112
SLC25A35	20.7658261	9.02417008	-1.2023451	9.69E-11	8.61E-09

Table S2 The enriched GO terms of differentially expressed genes.

ID	Category	GO Name	P.value	Count	geneID
GO:0007218	BP	neuropeptide signaling pathway	2.07E-06	7	NTS/NPW/NPY/CALCA/GAL/PNOC/PCSK1N
GO:0044306	CC	neuron projection terminus	1.41E-05	7	GRIK5/CALB2/UCHL1/NTS/CALCA/PNOC/CPLX2
GO:0043679	CC	axon terminus	6.26E-05	6	GRIK5/CALB2/NTS/CALCA/PNOC/CPLX2
GO:0043195	CC	terminal bouton	0.00025053	4	GRIK5/CALB2/CALCA/CPLX2
GO:0005184	MF	neuropeptide hormone activity	1.51E-08	6	NTS/NPY/CALCB/GAL/PNOC/VGF
GO:0005179	MF	hormone activity	3.02E-08	9	SST/ENHO/NTS/NPY/CALCB/CALCA/GAL/PNOC/VGF
GO:0001664	MF	G protein-coupled receptor binding	1.09E-07	12	RSPO3/CXCL5/UCHL1/NTS/NPW/NPY/CALCB/CCKBR/CALCA/GAL/PNOC/PF4V1
GO:0048018	MF	receptor ligand activity	2.00E-05	12	CXCL5/SST/ENHO/NTS/NPY/CALCB/GREM2/CALCA/GAL/PNOC/VGF/PF4V1
GO:0030546	MF	signaling receptor activator activity	2.34E-05	12	CXCL5/SST/ENHO/NTS/NPY/CALCB/GREM2/CALCA/GAL/PNOC/VGF/PF4V1
GO:0071855	MF	neuropeptide receptor binding	5.55E-05	4	NTS/NPY/CCKBR/GAL
GO:0005200	MF	structural constituent of cytoskeleton	0.00029792	5	NEFH/TUBA3E/TUBA3D/TUBA4B/TUBB2B
GO:0005201	MF	extracellular matrix structural constituent	0.00043942	6	COL9A3/FBN3/MUC5AC/GP2/MUC6/COMP
GO:0048306	MF	calcium-dependent protein binding	0.00157062	4	DMBT1/S100A1/TNNI3/CPLX2
